# Exploration of Bioactive Components, Nutritional Properties, Antioxidant, Antimicrobial and Antihemolytic Potential of Different Parts of *Mentha longifolia* L.

**DOI:** 10.1002/fsn3.70634

**Published:** 2025-07-27

**Authors:** Meryem Tourabi, Youness El Abdali, Khaoula Faiz, Ibrahim Mssillou, Abdelkrim Agour, Bouchra Louasté, Mohammed Merzouki, Ahmad Mohammad Salamatullah, Mohammed Bourhia, Youssouf Ali Younous, Amira Metouekel, Badiaa Lyoussi, Elhoussine Derwich

**Affiliations:** ^1^ Laboratory of Biotechnology, Conservation and Valorisation of Bioresources Faculty of Sciences, Sidi Mohamed ben Abdellah University Fez Morocco; ^2^ Laboratory of Biotechnology, Environment, Agri‐Food, and Health (LBEAS), Faculty of Sciences Dhar El Mahraz, Sidi Mohamed ben Abdellah University Fez Morocco; ^3^ Laboratory of Biotechnology, Environment, Agri‐Food and Health Faculty of Sciences, Sidi Mohamed ben Abdellah University Fez Morocco; ^4^ Department of Food Science and Nutrition College of Food and Agricultural Sciences, King Saud University Riyadh Saudi Arabia; ^5^ Laboratory of Biotechnology and Natural Resources Valorization Faculty of Sciences, Ibn Zohr University Agadir Morocco; ^6^ Evangelical College N'Djamena Chad; ^7^ University of Technology of Compiègne EA 4297 TIMR Compiegne Cedex France; ^8^ Unity of GC/MS and GC‐FID City of Innovation, Sidi Mohamed ben Abdellah University Fez Morocco

**Keywords:** antihemolytic potential, antimicrobial activity, antioxidant activity, HPLC‐DAD, *M. longifolia*, nutritional properties

## Abstract

*Mentha longifolia* is frequently utilized as a natural treatment in conventional medicine to treat diverse illnesses, including gastrointestinal, respiratory, and inflammatory disorders. The present study aimed to unveil the phenolic composition, as well as the antioxidant, antihemolytic, and antimicrobial potential of different parts of *Mentha longifolia* . HPLC‐DAD analysis was employed to identify the phenolic components of extracts from different plant parts. The total flavonoid and phenolic contents were measured. Four assays were conducted to assess the antioxidant capacity in vitro, including DPPH, ABTS, RP, and TAC assays. The antihemolytic potential of different parts of *M. longifolia* was also evaluated. The minimum inhibitory concentration (MIC) was determined using the microdilution method, and the minimum bactericidal concentration (MBC) was assessed. Thirteen compounds were identified in *M. longifolia* extracts, among them kaempferol, quercetin, ferulic acid, and gallic acid. However, the aerial part of *M. longifolia* (leaves and stem) and root extract contained a high polyphenol content and exhibited nutritional properties. Leaf extract comprises a high level of flavonoid content combined with significant antioxidant potency and demonstrated excellent antibacterial and antifungal activity. Moreover, a significant antihemolytic capacity was observed in the root, stem, and seed extracts. Based on the findings of the current study, *M. longifolia* extracts exhibited high antihemolytic power along with antioxidant and antimicrobial properties, suggesting their potential use in the development of plant‐based drugs in the future.

## Graphical abstract

*Mentha longifolia* extracts demonstrated significant antioxidant, antimicrobial, and antihemolytic properties, with high polyphenol and flavonoid content, particularly in the leaves and roots. HPLC‐DAD analysis identified 13 phenolic compounds, including kaempferol, quercetin, ferulic acid, and gallic acid, which were the major. Leaf extract exhibited strong antibacterial and antifungal activity, while root, stem, and seed extracts showed notable antihemolytic potential. These findings suggest that *M. longifolia* holds great potential for use in plant‐based food production and therapeutic applications.

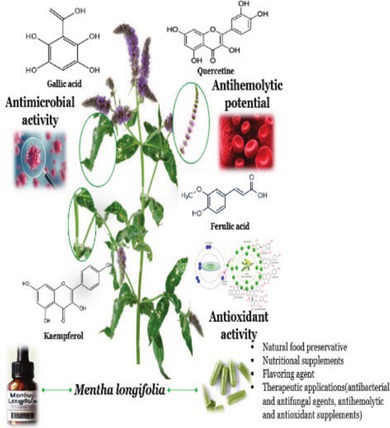

## 1 Introduction

Nowadays, medicinal plants play several pivotal roles in therapy, nutrition, and overall well‐being. They serve not only as key components in traditional and modern medicine but also as natural sources of nutrients in food. They are crucial for managing chronic disorders as well as for preventive health care, since their therapeutic qualities help treat a wide range of illnesses, including cancer, diabetes, gastrointestinal disorders, and respiratory conditions, among others.

*Mentha longifolia* L. is one of the most advantageous medicinal plants containing substantial bioactive secondary metabolites with interesting therapeutic potential (including antihemolytic, antimicrobial, anti‐inflammatory, anti‐proliferative, hepatoprotective, antidiabetic, gastroprotective, and antispasmodic properties) (Farzaei et al. 2017). It is a precious trove of native active metabolites, particularly due to its high concentrations of phenolic, terpenoids, and flavonoid components. *M. longifolia* offers an abundance of potent antioxidant substances, including rutin, kaempferol, quercetin, and gallic, caffeic, and rosmarinic acids (Tourabi, El Ghouizi, et al. 2023). Additionally, recent studies have stated that *M. longifolia* reduces tumor cell viability and exhibits anti‐quorum‐sensing activities (Haikal et al. 2021; Afkar and Somaghian 2024). Due to its recognized dietary benefits and medicinal attributes, *M. longifolia* is utilized as a flavoring agent in both raw and processed foods, including salads, soups, cheese, bread, and herbal teas (Anwar et al. 2019).

Nevertheless, only a limited number of studies have investigated the chemical composition and biological properties of various *M. longifolia* parts, which have been recorded. Thus, the purpose of the current study was to assess the antihemolytic characteristics, antioxidant ability, phytochemical profile, nutritional composition, and antimicrobial potential of different parts of *M. longifolia* from Morocco.

## 2 Result and Discussion

### 2.1 Extraction Yield

**FIGURE 1:**
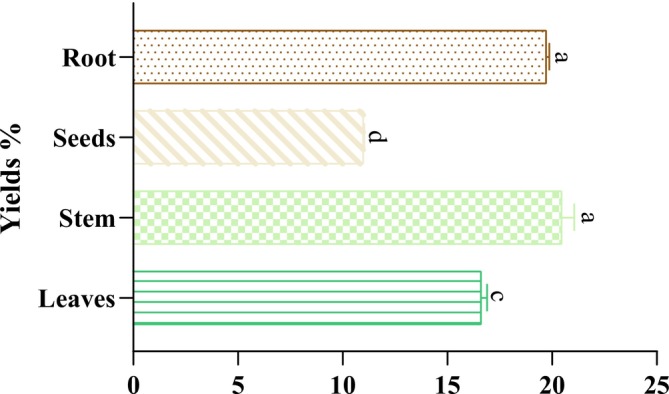
Dry weight extraction yields for various *M. longifolia* extracts. The values with the same letter in the same test are not statistically different (*p* > 0.05). Means values ± SD (*n* = 3).

The findings of the extraction yield of the different *M. longifolia* extracts are displayed in Figure 1. In addition, the percentage of extraction yield varied significantly among the plant parts and ranged from 10.99% ± 0.03% to 20.46% ± 0.59%, with the following decreasing order: root > stem > leaves > seeds extract. The highest percentage yield was observed in the root and stem (20.46% ± 0.59% and 19.72% ± 0.14%) Moreover, the seeds had a lower percentage yield, with a value of 10.99% ± 0.03%. The obtained data corroborate those found by El‐hawary et al., who reported that the highest extraction yield of *Plectranthus amboinicus* was observed in the root and stem parts, with a value of 12.5% and 7.5%, respectively. Indeed, several factors influence plant extraction yield, including the plant itself, the plant part used, harvesting practices, extraction methods, and environmental conditions (Olubode et al. 2016).

### 2.2 Phenolic Content and Constitution of Different *M. longifolia* Extracts

It has been stated that *M. longifolia* is a food rich in polyphenols which enhance health. Table 1 illustrates the different amounts of phenolic compounds found in the various parts of *M. longifolia* . Furthermore, the total polyphenol content of extracts from *M. longifolia* ranges from 56.30 ± 0.89 to 77.29 ± 0.11 mg GAE/g dw. The leaf extract had the greatest TPC (77.29 mg GAE/g DW) followed by 77.14 ± 0.40 mg GAE/g DW (root) and stem at 76.78 ± 0.76 mg GAE/g DW, while the extracts from seeds indicated low TPC. According to the literature review and to the best of our knowledge, there are no reports on the phenolic contents of various parts of *M. longifolia* extracts. However, the phenolic content in *Mentha* species has been studied. In this context, Farnaz Malik et al. investigated the secondary phytochemicals of *Mentha arvensis* harvested from Pakistan. The findings showed that all of the parts of *M. arvensis* , especially the leaf extracts, are rich in phenols, with a concentration of 3.51% ± 0.1%, followed by the roots extract with a concentration of 1.95% ± 0.03% (Malik 2012). The registered flavonoid content varied between 8.93 ± 0.17 and 38.80 ± 0.05 mg QE/g dw. The high level of flavonoids is recorded in the leaf extract (38.80 ± 0.05 mg QE/g dw), while the root extract contained the lowest level of flavonoids with a concentration of 8.93 ± 0.17 mg QE/g dw when it was less than that stated by Farnaz Malik et al. for a Pakistan *M. arvensis* (22.86% ± 0.3%) (Malik 2012). The phytochemical profiles of plant extracts were influenced by several factors, including the plant parts used, the extraction procedure, and the selection of solvent. The following could be the explanation for the observed variations in TPC and TFC throughout the analysis samples.

**TABLE 1:** Total phenolics (TPC) and flavonoids content (TFC), and phenolic components quantification of different *M. longifolia* extracts.

*M. longifolia* extracts	Leaves	Stem	Seeds	Root
TPC (mg GAE/g dw)	77.29 ± 0.11	76.78 ± 0.76	56.30 ± 0.89	77.14 ± 0.40
TFC (mg QE/g dw)	38.80 ± 0.05	16.67 ± 0.06	22.81 ± 0.17	8.93 ± 0.17
**Hydroxycinnamic acids (mg/g of crude extract)**				
Ferulic acid	2.12	4.19	2.00	1.58
Caffeic acid	0.99	0.75	0.85	0.83
Vanillic acid	0.05	0.23	0.98	0.50
Coumaric acid	0.80	0.12	ND	0.09
Rosmarinic acid	0.07	0.27	0.01	ND
**Hydroxybenzoic acids (mg/g of crude extract)**				
Syringic acid	0.45	0.25	0.17	0.17
Protocatechuic acid	1.64	2.11	0.38	0.20
Gallic acid	0.49	2.07	1.44	1.64
Pyrogallol	0.94	1.48	0.54	0.25
**Flavonoid (mg/g of crude extract)**				
Rutin	0.65	0.35	0.98	0.20
Quercetin	ND	3.69	1.47	1.90
Catechol	0.81	1.28	0.67	0.89
Kaempferol	0.28	3.84	0.18	0.19

Abbreviation: ND, non‐determined.

By employing HPLC‐DAD analysis, an average content, including hydroxycinnamic acid, hydroxybenzoic acid, and other flavonoids, was determined to be detected in every extract. The determination of individual phenolic composition was performed to emphasize how specific plant parts affect the chemical profile of *M. longifolia* and consequently its bioactivities. As shown in Table 1, quercetin was not found in leaf extracts and was identified in high amounts in other parts such as stem extract containing 3.69 mg/g of crude extract. However, kaempferol was the major flavonoid detected in the stem extract (3.84 mg/g of crude extract). Ferulic acid was the main phenolic compound detected in stem (4.19 mg/g of crude extract), leaves (2.12 mg/g of crude extract), seeds (2.00 mg/g of crude extract), and root (1.5 mg/g of crude extract), while gallic acid was the main compound found in stem (2.7 mg/g of crude extract). Rosmarinic acid was not identified in root extracts and was detected in lower concentrations in other parts of the extracts.

Indeed, various parameters/factors affect and suggest the broad variation in the phytochemical composition among the examined extracts, including the plant part, extraction technique, solvent polarity, method of extract preparation, quantification method, and identification of biomolecules as well as the environmental conditions. The significant variation in the phytochemical composition of samples indicates the presence of specific bioactive compounds in different plant parts. It is important to note that ferulic acid, protocatechuic acid, quercetin, and kaempferol were the major phenolic acids and flavonoid components identified in all studied samples. Thus, this diverse range of secondary metabolites enhances the antioxidant capacity of *M. longifolia* extracts, endowing it with a wide range of biological and pharmacological properties.

The phenolic compounds identified in *M. longifolia* are widely recognized for their beneficial biological activities, thus justifying the interest of this study in the medicinal applications of the plant. Ferulic acid and caffeic acid, a natural phenolic acid found in plant extracts, have a powerful antihemolytic efficiency by controlling human erythrocyte suspension and hemolysis caused by hypotonicity (Purba and Paengkoum 2022). It has also been previously stated that quercetin and kaempferol play preventive, antiproliferative, and anticancer roles (ex: endometrium, colon, esophagus, stomach, and lung cancers) by several molecular mechanisms (Kubina et al. 2021). The treatment of tongue cancer with quercetin increases the cleavage of PARP, caspase‐3, caspase‐9, and caspase‐8 as well as the diminution of apoptotic protein xIAP (Li et al. 2019). Quercetin and kaempferol have anti‐cancer, cardioprotective, and neuroprotective effects demonstrated by several pharmacological studies. Rosmarinic acid is well documented for its anti‐inflammatory and antioxidant properties, playing a key role in protecting against oxidative stress and chronic inflammatory diseases (Nadeem et al. 2019). Likewise, other biomolecules such as caffeic acid, gallic acid, protocatechuic acid, rosmarinic acid, and vanillic acid, among others, have also been established to have a wide spectrum of therapeutic effects, notably antioxidant activity, antidiabetic activity, antiulcer activity, cardioprotective capacity, anticancer property, neuroprotective activity, and anti‐inflammatory activity (Saibabu et al. 2015).

Thus, this HPLC‐DAD analysis provides essential data for the valorization of Moroccan *M. longifolia* as a natural source of bioactive biomolecules, reinforcing the importance of the results obtained for pharmaceutical research and ethnomedicine.

### 2.3 Antioxidant Activities

Natural antioxidants are crucial for maintaining good health and treating serious illnesses (Gupta and Sharma 2006). Compounds derived from plants, such as flavonoids and polyphenols, have significant antioxidant capabilities. These compounds can provide comparable advantages to humans when ingested since they have evolved in plants to combat oxidative stress (Bautista‐Hernández et al. 2021). To quantify multiple mechanisms of action, four approved in vitro tests, namely DPPH, ABTS, RP, and TAC, were applied to evaluate the antioxidant effect of the studied extracts.

**FIGURE 2:**
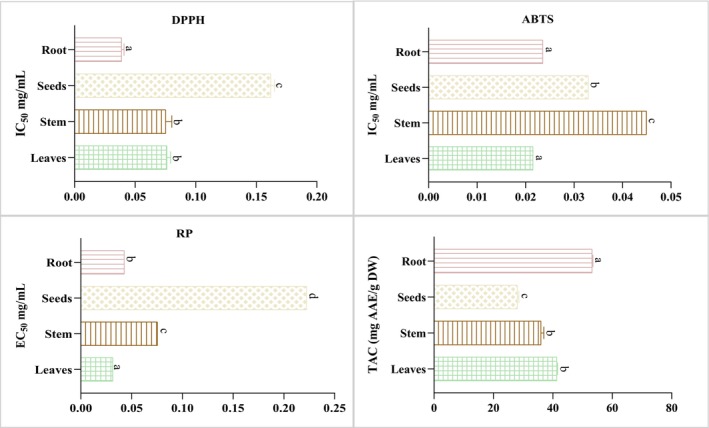
Antioxidant capacity of different *M. longifolia* extracts using DPPH, ABTS, reducing power test, and total antioxidant capacity. There is no statistically significant difference between findings from tests that have the same letter (*p* > 0.05).

The obtained results are illustrated in Figure 2 and show that all *M. longifolia* extracts exhibited potential antioxidant activity, while there were notable variances among them. For the DPPH and ABTS test, leaf and root extracts exhibited the highest antiradical capabilities, with IC_50_ of 0.076 ± 0.003 mg/mL and 0.03 ± 0.002 mg/mL for the DPPH test, and 0.021 ± 0.00 and 0.02 ± 0.00 mg/mL for the ABTS assay, respectively. This outcome is consistent with those achieved by Tourabi, Metouekel, et al. (2023), suggesting that the leaves of *M. longifolia* demonstrate strong antiradical capacities. The current research found that the stem and seed of *M. longifolia* have lower antioxidant capacity, regardless of the tests (DPPH and ABTS) that were performed. The effective reduction of power efficiency varies between extracts. Leaf and root extracts recorded high reducing power, with EC_50_ values of 0.03 ± 0.00 and 0.04 ± 0.00 mg/mL, respectively, whereas seed extract exhibited lower reducing power activity. These findings are greater than those stated previously by Dar et al. for the root of *Mentha arvensis* extracts, in which the EC_50_ value is 1.48 mg/mL (Dar et al. 2014).

Regarding total antioxidant capacity, the root extract showed a high total antioxidant capacity (53.21 ± 0.27 mg AAE/g DW), followed by the leaf extract (41.37 ± 0.19 mg AAE/g DW). The seed extract demonstrated a lower total antioxidant capacity (28.11 ± 0.24 mg AAE/g DW). The extracts possessed an antioxidant activity profile similar to total phenolic content, with the leaves and root parts exhibiting the greatest levels overall. This may justify the evident positive correlation between the antioxidant ability of the tested extracts and their phenolic compounds and content, which is confirmed by various other studies (Piluzza and Bullitta 2011; Turumtay et al. 2014).

### 2.4 Nutritional Quality Assessment

#### 2.4.1 Soluble Protein Content

**FIGURE 3:**
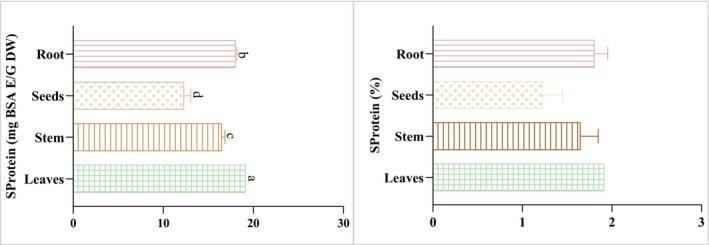
The soluble protein content of different *M. longifolia* extracts. There is no statistically significant difference between findings from tests that have the same letter (*p =* 0.05).

Protein is a crucial macronutrient found in various foods, playing an essential role in building and repairing tissues in the body. Furthermore, plant proteins have garnered attention for their potential role as functional foods (Hertzler et al. 2020). The food industry is particularly interested in plant‐based proteins due to their higher nutritional value compared to animal‐derived proteins. The soluble proteins of the studied extracts ranged between 12.28 ± 0.77 and 19.13 ± 0.00 mg BSAE/g DW. Moreover, the data obtained are lower than those of *Mentha piperita* leaves (9.80%) (Nonyelum 2023). In this context, leaf and root extracts have great potential for the food industry, as having a high content of soluble proteins is crucial to achieving optimal functionality in food processing applications (Figure 3).

#### 2.4.2 Soluble Carbohydrate Content

**FIGURE 4:**
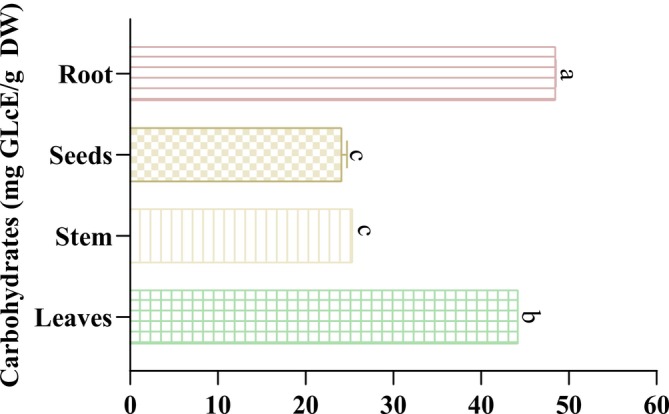
The soluble carbohydrate content of different *M. longifolia* extracts. There is no statistically significant difference between the outcomes of tests that have identical symbols (*p* > 0.05).

Among the many different types of nutrients, carbohydrates are the body's primary supply of energy. They are found in a wide array of foods and have various functions, which include metabolic function, energy production, and storage. Following the obtained findings (Figure 4), the soluble carbohydrate content varied significantly among extracts from 24.10 ± 0.63 to 48.48 ± 0.04 mg GlucE/g DW. Additionally, the root extract has a high content of soluble carbohydrates (48.48 ± 0.04 mg GlucE/g DW) followed by the leaves extract with a value of 44.23 ± 0.00 mg GlucE/g DW. Moreover, the seed extract has a lower soluble carbohydrate content (24.1 ± 0.63 mg GlucE/g DW). One study stated that the leaves of *Mentha piperita* contain high levels of soluble carbohydrates (34.13%). These findings highlight the potential of different parts of *M. longifolia* as sources of carbohydrates, which play a crucial role in metabolism, energy production, and storage.

In the context of food and nutrition, plant extracts rich in soluble carbohydrates, such as those from roots and leaves, can be utilized as natural energy sources in functional foods, dietary supplements, and nutraceutical formulations. Their incorporation into food products could enhance energy availability and contribute to balanced nutrition, particularly in health‐promoting diets.

### 2.5 Antimicrobial Potential

#### 2.5.1 Antibacterial Capacity

The antibacterial potential of all tested extracts is displayed in Table 2. The result shows that Gram‐negative bacteria ( *P. aeruginosa* and *E. coli*) are more sensitive to all tested extracts. The MIC values oscillate from 0.195 ± 0.01 to 0.391 ± 0.03 mg/mL for *E. coli* and 12.50 ± 0.04 mg/mL for *P. aeruginosa*, and aureus respectively. However, the different *M. longifolia* extracts showed a moderate inhibition of *B. subtilis* and *S. aureus* growth. The leaf and seed extract demonstrated the highest antibacterial activity against *E. coli* , showing a MIC value of 0.195 ± 0.01 mg/mL, which was more potent than ampicillin (the positive control) which had a MIC value of 0.626 ± 0.01. These outcomes are in harmony with those stated by Park et al. for the Korean Mint (Park et al. 2019). Furthermore, the seed, stem, and root extracts demonstrated that the ratio of MBC/MIC was lower than 4, suggesting a powerful bactericidal performance on the bacteria *P. aeruginosa* and *E. coli* . Additionally, the powerful antibacterial efficiency is correlated with the presence of the main constituents, especially kaempferol, quercetin, gallic acid, and ferulic acid, which have been shown previously to possess antibacterial capabilities (Aldulaimi 2017).

**TABLE 2:** Antibacterial potential of different *M. longifolia* extracts (mg/mL).

	Gram‐negative bacteria								Gram‐positive bacteria							
	*P. aeruginosa*				*E. coli*				*B. subtilis*			*S. aureus*				
	MIC	MBC	MBC/MIC	Effect	MIC	MBC	MBC/MIC	Effect	MIC	MBC	MBC/MIC	Effect	MIC	MBC	MBC/MIC	Effect
Leaves	12.50 ± 0.04	ND	—	—	0.195 ± 0.01	ND	—	—	6.250 ± 0.12	ND	—	—	6.250 ± 0.11	12.666	12.666	Bstatic
Stem	12.50 ± 0.05	25.000	2	Bcidal	0.391 ± 0.2	ND	—	—	6.250 ± 0.10	ND	—	—	6.250 ± 0.09	ND	—	—
Seeds	12.50 ± 0.4	25.000	2	Bcidal	0.195 ± 0.02	ND	—	—	6.250 ± 0.06	ND	—	—	6.250 ± 0.04	16.666	16.666	Bstatic
Root	12.50 ± 0.07	50.000	4	Bcidal	0.391 ± 0.03	ND	—	—	12.500 ± 0.30	ND	—	—	12.500 ± 0.12	50	50	Bstatic
SPM	0.630 ± 0.01	0.312	0	Bcidal	—	—	—	—	—	—	—	—	—	—	—	—
AMP	—	—	—	—	0.626 ± 0.01	0.317	0	Bcidal	0.315 ± 0.01	0.315	1	Bcidal	0.626 ± 0.01	0.158	0	Bcidal

Abbreviations: AMP, ampicillin; Bcidal, bactericidal; Bstatic, bacteriostatic; MBC, minimal bactericide concentration; MIC, minimal inhibitory concentration; ND, non determined; SPM, streptomycin.

The mechanisms of action of phenolic compounds have attracted a large amount of interest from researchers. Thus, several researchers have stated the antibacterial capacity of phenolic constituents (gallic acid, and ferulic acid) (Borges et al. 2013; Keyvani‐Ghamsari et al. 2023). Nevertheless, gallic acid (GA) and ferulic acid (FA) caused irreversible modifications in the characteristics of the membrane (charge, intra and extracellular permeability, and physicochemical properties) via alterations to hydrophobicity, a decrease in surface charge, leakage of vital intracellular components, and the development of local breakage or creation of pores in the cell membranes (Borges et al. 2013). Other phytoconstituents have also been indicated as having antibacterial capability, notably kaempferol and quercetin. Quercetin interacts with the bacterial cell and blocks bacterial growth by facilitating oxidative cellular stress, as well as by reducing the availability of L‐tryptophan in the local area via activating the kynurenine pathway (Adeyemi et al. 2020). Other reports confirmed that kaempferol had a strong antibacterial potential through several molecular pathways, namely causing bacterial damage to cells, severe alterations in the architecture of cell walls, plasmolysis causing excretion of intracellular constituents, and showing a decrease in the fluidity and integrity of cell membranes (He et al. 2014).

#### 2.5.2 Antifungal Potential

The in vitro antifungal capacity assay is displayed in Table 3, and the results show that all extracts except root extract exhibited the same antifungal capacity against both fungi ( *C. albicans* and *A. niger* ) with MIC values of 6.25 mg/mL and MFC values ranging between 16.66 and 25.00. The leaf, stem, and seed extracts had a ratio of MFC/MIC less than 4, suggesting a fungicidal effect against *C. albicans* and *A. niger* . In the same context, several reports have demonstrated the antifungal capacity and mechanisms of phenolic acids and flavonoid compounds, notably gallic acid, caffeic acid, kaempferol, and quercetin against pathogenic fungi (Teodoro et al. 2015; Al Aboody and Mickymaray 2020). Gallic acid is recognized to have an antifungal action against *C. albicans* (MTCC183) by affecting the *Candida* cytoplasmic layer, causing a modification in the charge and hydrophobicity of the cell wall, which eventually leads to the discharge of cytoplasmic contents. It was previously proposed that caffeic acid and its derivatives might have similar impacts versus *C. albicans* (ATCC10231) (De Vita et al. 2014). Another research stated that protocatechuic acid exhibited an anticandidal effect against *C. albicans* (LMP709U) with a MIC = 156 (μg/ml) and MFC = 312 (μg/ml) (Kuete et al. 2009). Besides, prior research has further revealed the strong antifungal potency and mechanisms of action of flavonoid components including quercetin and kaempferol. They commonly inhibit fungal growth through different underlying mechanisms by altering the plasma membrane and function of mitochondria, as well as inhibiting cell barrier formation, cell division, and synthesis of proteins and RNA (Al Aboody and Mickymaray 2020).

**TABLE 3:** Antifungal potential of various *M. longifolia* extracts (mg/mL).

	*C. albicans*				*A. niger*			
	MIC	MFC	MFC/MIC	Effect	MIC	MFC	MFC/MIC	Effect
Leaves	6.25 ± 0.04	25.00	4	Fungicidal	6.25 ± 0.01	—	—	—
Stem	6.25 ± 0.02	25.00	4	Fungicidal	6.25 ± 0.00	—	—	—
Seeds	6.25 ± 0.08	16.66	2.66	Fungicidal	6.25 ± 0.10	16.66	2.66	Fungicidal
Root	0.00 ± 0.00	ND	—	—	0.00 ± 0.00	ND	—	—
FCZ	1.30 ± 0.10	6.30 ± 0.01	4.84	Fungicidal	1.25 ± 0.04	6.30 ± 0.00	5	Fungistatic

Abbreviations: FCZ, fluconazole; MFC, minimal fungicide concentration; ND, non determined.

### 2.6 In Vitro Antihemolytic Capacity

**FIGURE 5:**
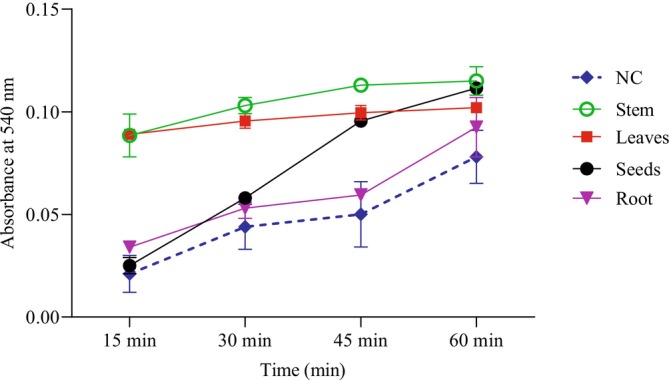
Evolution of absorbance at 540 nm of *M. longifolia* extracts (at 1.56 mg/mL concentration) and negative control (NC) in the hemolysis test during 60 min.

A hemolysis test was conducted on *Mentha longifolia* extracts to evaluate their biocompatibility and potential safety regarding red blood cells. This in vitro cytotoxic assay assesses the ability of the extract's compounds to disrupt erythrocyte membranes, leading to the release of hemoglobin due to cell lysis (Atta et al. 2023). This experiment tested extracts from different parts of *M. longifolia* on rat red blood cells. At a concentration of 1.56 mg/mL, the results (Figure 5) demonstrate a progressive increase in absorbance levels measured at 0, 15, 30, and 60‐min intervals compared to the negative control. This time‐dependent increase in absorbance correlates directly with an elevated rate of cell lysis, indicating a proportional rise in hemolytic activity with longer exposure times. Comparatively, the leaves and stem extracts exhibited a more pronounced hemolytic activity on red blood cells, whereas no significant difference (*p* > 0.05) in hemolytic effect was observed between the root extract and the negativecontrol.

**FIGURE 6:**
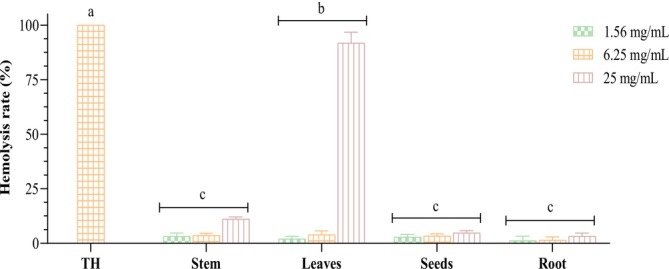
Hemolytic rate (%) of various concentrations of *M. longifolia* extracts after 60 min of incubation compared to total hemolysis (TH). Bars of different plant parts with different letters represent statistically different values (*p* < 0.05).

To assess the effect of concentration on the hemolytic activity of *M. longifolia* , the hemolysis rate (%) induced by the extracts at varying concentrations from different parts of the plant was evaluated relative to the total hemolysis observed. The results, presented in Figure 6, reveal that concentrations ranging from 1.56 to 25 mg/mL resulted in hemolysis rates between 2.86% ± 1.18% and 4.73% ± 1.03% for the seed extract, and from 3.16% ± 1.51% to 11.05% ± 0.99% for the stem extract of the studied plant in a dose‐dependent manner. The root extract demonstrated low hemolytic activity compared to the other parts, with a hemolysis rate not exceeding 3.20% ± 1.47% even at the highest concentration of 25 mg/mL. In contrast, the application of the leaf extract at the same concentration produced a significantly elevated hemolysis rate when contacted with rat erythrocytes, reaching up to 91.77% ± 4.93%. Additionally, all extracts across the various concentrations exhibited statistically lower hemolysis rates (*p* < 0.05) when compared to the positive control. These outcomes underscore the lower cytotoxicity and therefore higher biocompatibility of the roots, seeds, and stem extracts of *M. longifolia* with normal tissues. However, caution is necessary when applying the leaf extract, particularly at concentrations exceeding 6.25 mg/mL, due to its pronounced hemolytic effects.

Erythrocytes, the most abundant cells in the human body, exhibit unique biological and morphological properties but lack the replication capacity. As central mediators of redox‐active oxygen transport, they interact extensively with hemoglobin and polyunsaturated fatty acids. This makes them particularly susceptible to oxidative damage, where lipid and protein oxidation within the erythrocyte membrane can trigger hemolysis. Several factors contribute to this oxidative stress, including impaired antioxidant defense mechanisms within erythrocytes, radiation exposure, elevated concentrations of transition metals, oxidizing agents, and underlying hemoglobinopathies (Hamidi and Tajerzadeh 2003; Karim et al. 2020).

The hemolysis test serves as a key indicator of cytotoxicity by assessing the degradation of red blood cells when exposed to varying concentrations of a natural extract. This method is critical for evaluating the cytotoxic effects of such extracts, thereby playing a significant role in advancing the fields of phytotherapy and pharmacological formulation. Typically, the hemolytic activity of a natural product or drug can arise through different mechanisms, including membrane disruption, increased permeability, or complete cell lysis (Sayes et al. 2007).

The hemolytic ability of *Mentha longifolia* extracts has been explored in a few studies, with a focus on their cytotoxicity toward red blood cells, antioxidant properties, and capacity to protect against hemolysis provoked by oxidative stress. In this regard, recent research stated that the crude extract of *M. longifolia* exhibited a low hemolytic effect, with a dose‐dependent decline. Specifically, hemolysis rates of 9.13%–2.74%, 9.56%–2.31%, and 10.42%–3.17% were observed at concentrations of 200, 100, 50, and 25 μg/mL for ethyl acetate, methanol, and aqueous extracts, respectively (Atta et al. 2023). Additionally, research on the aerial parts of *M. longifolia* showed that the hydroethanolic extract exhibited antihaemolytic activity, particularly against hydrogen peroxide‐induced hemolysis. The concentration required to inhibit 50% hemolysis (IC_50_) was determined to be 951.4 μg/mL, indicating its protective effect against oxidative damage to red blood cells, which is crucial for maintaining cellular integrity under stress conditions (Ebrahimzadeh et al. 2010). Moreover, a recent study revealed that silver nanoparticles phytosynthesized from *M. longifolia* aqueous extract were biocompatible with human red blood cells at lower doses, with an LD_100_ recorded at 117 μg/mL (Javed et al. 2020).

To the best of our knowledge, this is the first research assessing the hemolytic effects of extracts from different parts of *M. longifolia* . Our findings highlight the lower cytotoxicity of the roots, seeds, and stem extracts of *M. longifolia* on rat red blood cells, aligning with previous studies. In general, phytochemicals can influence the stability of red blood cell membranes, depending on their antioxidant profiles and the potential interactions of specific functional groups with the cell membrane (Rubnawaz et al. 2021). The observed protective effect of *M. longifolia* extracts, particularly at lower concentrations, is likely due to the presence of various phenolic metabolites found in the plant. Previous research has indicated that phenolic compounds, known for their potent antioxidant properties, play a significant role in preserving erythrocyte membranes from oxidative damage, thereby reducing susceptibility to hemolysis (Ali et al. 2018). This is the case for flavonoids such as quercetin, rutin, and kaempferol, abundant in the studied extracts, which have demonstrated significant antihaemolytic activity in a dose‐dependent manner when red blood cells were subjected to oxidative stress (Asgary et al. 2005; Olchowik‐Grabarek et al. 2023). Similarly, phenolic acids such as caffeic, gallic, syringic, and coumaric acids, detected in the extracts, have also been reported and recognized for their potential to protect red blood cells against hemolysis induced by oxidative stress (Abdel Moneim et al. 2018; Chanda and Juvekar 2018; Oršolić et al. 2021).

Conversely, the pronounced hemolytic effect observed at higher concentrations of *M. longifolia* leaf extract may be attributed to the presence of certain metabolites or compounds with known hemolytic properties, such as saponins, as reported by (Adham 2015; Zeenat Waris et al. 2018). These substances interact with cholesterol in erythrocyte membranes, increasing their permeability and ultimately leading to hemolysis (Andleeb et al. 2020). Additionally, some phytochemicals can exert dual effects on the integrity of red blood cells depending on concentration. The literature suggests that higher concentrations of tannic acid, for instance, can induce crinkling and lysis of red blood cells (Deng et al. 2019). In general, the hemolytic effect of any compound or molecule is influenced by several factors, including temperature, incubation time, the presence of side chains like saponins, and the specific composition of the cell membrane (Andleeb et al. 2020; Urbańska et al. 2009).

Although the studied extracts, particularly at low concentrations, exhibited minimal toxicity using in vitro tests, it remains essential to assess their interaction with biological systems through in vivo models. Furthermore, higher concentrations of *M. longifolia* leaf extract should be evaluated in cancer cell models to investigate its potential anticancer activity.

### 2.7 Statistical Analysis

#### 2.7.1 Correlation Pearson test

**FIGURE 7:**
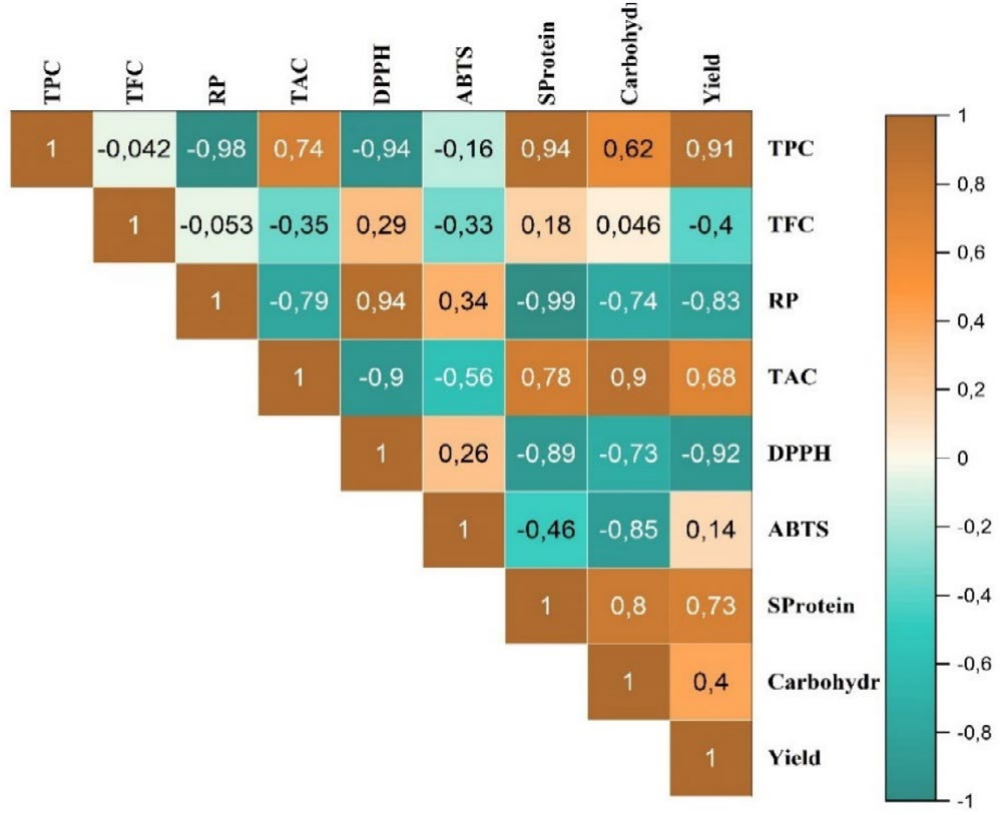
Correlation coefficient of the antioxidant capacity (DPPH, ABTS, RP, and TAC), total phenolic and flavonoid content, SProtein, soluble carbohydrates, and yield of different *M. longifolia* extracts using Pearson correlation assay.

The relationship among antioxidant activity, flavonoid content, total phenolic content, soluble proteins, soluble carbohydrates, and yields of different extracts of *M. longifolia* was assessed. Based on the data illustrated in Figure 7, the Pearson correlation analysis revealed positive and negative correlations. The negative correlation coefficient was found among the TPC level and antioxidant capacity (RP, and DPPH) with *r* ^2^ = −0.98 and *r* ^2^ = −0.94, respectively. However, TPC, TAC, soluble protein, and yield showed positive correlations. Furthermore, various researchers have previously studied the correlation analysis between TPC and the antioxidant potential of plant extracts (Maina et al. 2021; Idoudi et al. 2023).

#### 2.7.2 Principal Component Analysis

**FIGURE 8:**
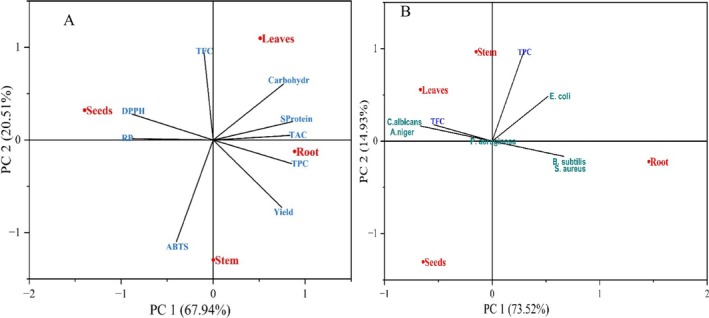
Principal component analysis (PCA). (A) The plot indicates the correlations between yield, antioxidant activity (DPPH, ABTS, RP, and TAC), TPC, TFC, SProtein (soluble protein), and carbohydrates (carbohydrates) of different *M. longifolia* extracts. (B) Plot showing the effect of different parts of *M. longifolia* on the antimicrobial activity.

To assess the influence of different parts of *M. longifolia* on the TPC, TFC, antioxidant capability, yield, SProtein, soluble carbohydrates, and antimicrobial efficiency, the principal component test was conducted, and Figure 8A,B show the main results. Figure 8A indicates the principal components that include TPC, TFC, antioxidant activity, yield, SProtein, and soluble carbohydrates of different parts of *M. longifolia* . The majority of the observed variance was captured by the first two principal components, with PC1 explaining 67.94% and PC2 accounting for 20.51%. The PC1 vs. PC2 plot shows a significant negative correlation between TPC and antioxidant ability (DPPH, ABTS, and RP), where it is significantly correlated with SProtein and soluble carbohydrates. This indicates that extracts exhibiting the highest antioxidant capacity also contained great levels of total phenolic content. Moreover, TPC, TAC, carbohydrate, and SProteins were regrouped positively with root extract, whereas DPPH and RP assay regrouped negatively with root extract and positively with seed extract.

Figure 8B demonstrates the PCA for the level of phenolic and flavonoid as well as the antimicrobial ability of different part extracts of *M. longifolia* , with the variation of PC1 (87.86) and PC2 (12.14%). TFC was correlated negatively with *E. coli* , *B. subtilis*, and *S. aureus* , whereas TPC had a negative correlation with *C. albicans* and *A. niger* . The variables (TFC, TPC) had a positive relationship with leaf and stem extracts and an adverse correlation with root extract. It was the explanation that the leaf and stem extracts had a high level of phenolic and flavonoid compounds/content and also had a potent antimicrobial capacity.

## 3 Material and Methods

### 3.1 Botanical Matrices and Extract Preparation

Samples were gathered in the localities of Ifrane in May 2021. The voucher specimen is deposited in the faculty herbarium, with the identification number 001MLAV202162. To learn more details, please visit the article (Tourabi, Metouekel, et al. 2023). Briefly, 1 g of powder from each part (leaves, stem, seeds, and root) of *M. longifolia* and 10 mL of 70% v/v hydroethanolic solution were mixed, and the mixture was macerated and continually shaken at the ambient temperature for 1 week. After the extracts had been purified of particles by applying a Whatman N°1 filter, they were concentrated at 40°C in a rotating vacuum evaporator. Before being used, the resultant crude extracts were gathered and kept at −20°C.

### 3.2 HPLC‐DAD Examination

The phytochemical profile of different parts of *M. longifolia* was evaluated using a High‐performance Liquid Chromatography Diode Array Detector (HPLC‐DAD) according to the protocol achieved by Tourabi, El Ghouizi, et al. (2023). The extracts (10 mg) were dissolved in 1 mL of 80% methanol and filtered through 0.45 mm filters. Phenolic compounds were separated using a Wakosil C18HG column (5 mm, 4.6 × 150 mm) at a controlled temperature of 40°C. The elution process was carried out in gradient mode using a binary solvent system: solvent A consisted of water acidified with 0.2% phosphoric acid, while solvent B was a 50/50 mixture of methanol and acetonitrile. The gradient started at 96% (A) and 4% (B), transitioning to 50% (A) and 50% (B) over 40 min. It then shifted to 40% (A) and 60% (B) for 5 min before reaching 0% (A) and 100% (B) for 15 min. Finally, the system was re‐equilibrated to its initial composition for 12 min. At a flow rate of 1 mL/min, each sample was injected with a volume of 20 μL into the mobile phase. The standard components, namely ferulic acid, caffeic acid, vanillic acid, coumaric acid, rosmarinic acid, syringic acid, protocatechuic acid, gallic acid, pyrogallol, rutin, quercetin, catechol, kaempferol, were dissolved in methanol of HPLC grade. The phenolic compounds were determined using the reference compounds using retention times and absorption spectra.

### 3.3 Assessment of Total Phenolic Content

The Folin–Ciocalteu technique was used to quantify the total phenolic content according to the protocol indicated by Tourabi, El Ghouizi, et al. (2023). Briefly, a 500 μL aliquot of Folin solution (diluted 1:10) was combined with 50 μL of each sample. After a 5‐min incubation, 400 μL of a 7.5% sodium carbonate solution (Na_2_CO_3_) was added. The mixture was then incubated in the dark for 2 h, and the absorbance was measured at 760 nm. Gallic acid was employed as a standard to create the calibration graph (R^2^ = 0.996). The total phenolic amount (TPC) was expressed as (mg GAE/g DW).

### 3.4 Assessment of Total Flavonoid Content

The colorimetric assay of aluminum chloride (AlCl_3_) was used to estimate the level of flavonoid in the studied extracts according to the protocol outlined by Tourabi, El Ghouizi, et al. (2023). In brief, 500 μL of a 10% AlCl_3_ solution was added to 500 μL of each sample and standard. After incubating for 1 h in the dark, the absorbance was measured at 420 nm. As a standard, we used Quercetine to establish the standard curve (*R* ^2^ = 0.994), and the TFC values were provided in (mg QE/g DW).

### 3.5 In Vitro Antioxidant Power

To assess the antioxidant ability, four protocols were used, including the DPPH test, ABTS assay, reducing power (RP), and total antioxidant capacity (TAC).

#### 3.5.1 DPPH Scavenging Capacity

The free radical scavenging activity of each extract was assessed using the 1,1‐diphenyl‐2‐picrylhydrazyl (DPPH) method, as described by Tourabi, Metouekel, et al. (2023). Briefly, 50 μL of each extract was mixed with 825 μL of a DPPH solution (6 mol/L in ethanol), adjusted to an initial absorbance of 0.7 at 517 nm. The reaction mixture was incubated at room temperature for 1 h, after which the absorbance was measured at a wavelength 517 nm (UV–Vis spectrophotometer). The IC_50_ values were determined from the inhibition percentage (IP) plotted against concentration, using the following formula (Equation 1):

(1) $$\mathrm{IP}\left(\%\right)=\left(\frac{\text{Absorbance of control}-\text{Absorbance of sample}}{\text{Absorbance of control}}\times 100\right)$$

#### 3.5.2 ABTS Decolorization Assay

The 2,2′‐azino‐bis (3‐ethylbenzothiazoline‐6‐sulfonic acid) radical cation‐based (ABTS test) was evaluated as outlined in the protocol cited by Ferreira‐Santos et al. (Ferreira‐Santos et al. 2020). A total of 825 μL of 2,2′‐azino‐bis (3‐ethylbenzothiazoline‐6‐sulfonic acid) diammonium salt (ABTS) at 7 mM was added to 50 μL of each extract. After incubating the mixture in the dark for 30 min, the absorbance was measured at 734 nm using a UV/Vis spectrophotometer. The percentage of ABTS inhibition was determined using the following formula (Equation 2):

(2) Inhibition%=Ac−AsAc×100

where *Ac* represents the absorbance of the control and *As* represents the absorbance of the sample. All measurements were performed in triplicate, and the results were expressed in mg/mL.

#### 3.5.3 Reducing Power Assay (RP)

The reducing power of the extracts was evaluated following the method described by Tourabi, Metouekel, et al. (2023) In brief, 100 μL of *M. longifolia* extracts were mixed with 500 μL of 0.2 M sodium phosphate buffer (pH 6.6) and 500 μL of 1% potassium ferricyanide. Afterward, 500 μL of 10% TCA was added, and the mixture was incubated at 50°C for 20 min. The absorbance was measured at 700 nm using a PerkinElmer Lambda 40 spectrophotometer. The results were expressed in EC_50_ as determined from the absorbance curve (*Y* = *ax* + *b*; *Y* = 0.5) and reported in mg/mL.

#### 3.5.4 Total Antioxidant Capacity

The total antioxidant capacity (TAC) of *M. longifolia* extracts was assessed based on the protocol described by Tourabi et al. For this, 1 mL of reagent solution (sulfuric acid 6 M, sodium phosphate 28 mM, and ammonium molybdate 4 mM) was added to 0.5 mL of each sample or standard (Ascorbic acid). After incubating the sample in a heated bath at 95°C for 90 min, its absorbance was measured at 700 nm using a PerkinElmer Lambda 40 UV/Vis spectrophotometer (Barcelona, Spain), with the blank as the reference. The absorbance values were expressed as milligrams of ascorbic acid equivalent per gram of extract (mg AAE/g DW), based on the standard curve (*Y* = 2.9010*x* + 0.0998, *R* ^2^ = 0.9996).

### 3.6 Nutritional Assessment

#### 3.6.1 Soluble Proteins Content

The approach provided by Lowry et al. was used to estimate the soluble protein amount (Oh 1951), with some slight modifications. In brief, 100 μL of each extract was combined with 1 mL of Lowry coloring reagent, and 100 μL of Folin reagent (1/3) was added. The mixture was placed in the dark for 30 min before using a spectrophotometer adjusted to measure absorbances at 750 nm. Bovine serum albumin (0.002–0.116 mg/mL) was employed as a standard for preparing a calibration curve (*R* ^2^ = 0.9906) and the results were given in milligrams (mg BSAE/g DW), or BSA equivalents per gram of dry matter.

#### 3.6.2 Total Soluble Carbohydrate Content

The phenol‐sulfuric acid procedure outlined by Masuko et al. (2005) was used to measure the total carbohydrate content. To proceed, 150 μL of sulfuric acid (96%–98% (v/v)) was added to 50 μL of each sample. Next, 30 μL of the phenolic reagent (5%) was added, and the mixture was heated to 90°C for 5 min. After cooling for 5 min to ambient temperature, the absorbance was measured using a spectrophotometer at a wavelength of 490 nm. The calibration curve was created using glucose as the reference standard (*R* ^2^ = 0.992). The total carbohydrate content was expressed as milligrams of glucose equivalents (GlcE) per gram of dry plant material (mg GlcE/g DW).

### 3.7 Antimicrobial Capacity

#### 3.7.1 Tested Microorganisms

The antibacterial and antifungal capacity of different parts (leaves, stem, seeds, and root) of *M. longifolia* was explored against four multi‐resistance pathogens (2 g‐positive and 2 g‐negative bacteria) and two fungi. To learn more details, please visit the article (Tourabi and Baghouz 2024).

#### 3.7.2 Assessing the Low Inhibitory Concentration (MIC)

The minimal inhibitory concentration of the samples (leaves, stem, seeds, and root) was determined in sterilized microplates employing the microdilution method according to the research stated by Tourabi, Metouekel, et al. (2023).

#### 3.7.3 Assessing the Low Bactericide and Fungicide Concentration (MBC/MFC)

The MBC and MFC are antimicrobial concentrations below which less than 0.01% of microbes survive. After identifying the MIC values, each well that shows no bacterial or fungal growth is analyzed, and the samples are then placed on Muller‐Hinton (MH) agar for bacteria and Sabouraud Dextrose Agar (SDA) for fungi. Following that, the agar plates are incubated at 37°C and 30°C, respectively. After a 24‐h incubation time, the MBC and MFC values were determined (Mirelle et al. 2016). In addition, an evaluation was conducted on the MBC/MIC and MFC/MIC ratios to determine the possible mechanism of the examined extracts/antibiotics.

### 3.8 Antihemolytic Capacity

#### 3.8.1 Blood Cell Preparation

Freshly collected rat blood samples were mixed with a heparin‐based anticoagulant solution. To obtain a pure erythrocyte suspension, the blood was subjected to three consecutive washes with sterile 0.9% NaCl saline solution. After each wash, the cells were separated by centrifugation at 3500 rpm for 10 min at 4°C, followed by careful aspiration of the supernatant. The erythrocytes were then resuspended in a physiological solution to create a 3% erythrocyte suspension, which was used in the hemolytic assay.

#### 3.8.2 Hemolytic Assay

The hemolytic activity of the examined plant was evaluated using an adjusted protocol based on the method outlined by (Saleh et al. 2021). In hemolysis tubes, 100 μL of three concentrations of each extract (1.56, 6.25, and 25 mg/mL) were mixed with 1900 μL of the pre‐prepared erythrocyte suspension. The mixtures were incubated at 37°C for 1 h in a water bath. Samples (500 μL each) were taken at 15‐min intervals over 60 min and then mixed with 1.5 mL of phosphate‐buffered saline (PBS). The tubes were subsequently centrifuged at 3000 rpm for 10 min. The absorbance of the supernatants, indicative of hemoglobin release due to erythrocyte lysis, was measured at 540 nm using a UV–visible spectrophotometer, with PBS serving as the blank. Under the same conditions, a negative control was established using PBS instead of the extract.

The percentage of hemolysis was determined relative to a total hemolysis tube, where distilled water replaced the extract under the same conditions. The hemolysis rate of the extract samples was computed following a 60‐min incubation period as a percentage of total hemolysis, using the following equation:

$$\text{Hemolysis rate}\;\left(\%\right)=\left[\left(A–A_{0}\right)/\left(A_{t}–A_{0}\right)\right]\times 100$$

Where A, A_0_, and A_t_ were, respectively, the absorbance of the sample, the absorbance of the negative control, and the absorbance of the positive control (total hemolysis).

#### 3.8.3 Statistical analysis

All experiments were independently performed three times, and the results are expressed as the average value with corresponding standard deviations. Statistical analyses were executed using GraphPad Prism 8.0.2 (263) software, applying a one‐way ANOVA followed by Tukey test for comparison, with significance considered at *α* = 0.05. The correlation coefficient tests and Principal Component Analysis (PCA) were carried out using OriginPro 2025 software (OriginLab Corporation, Northampton, MA, USA).

## 4 Conclusion

The data from the current work provide insights into the phenolic profile, nutritional composition, and bioactivities of different parts of *M. longifolia* from Morocco. The examined extracts have distinct properties compared to each other, potentially due to their phytochemical and nutritional composition. The findings gathered in the present examination show that the studied extracts contain a considerable amount of soluble proteins and carbohydrates, which are the major and essential macronutrients. The results revealed a strong phytochemical and nutritional profile, along with significant antioxidant, antihemolytic, and antimicrobial properties, attributed to the presence of various phenolic compounds. Generally, the correlation between the biochemical properties and nutritional characteristics indicates their potential application as a beneficial food source for fighting malnutrition and combating microbial infection, as well as preventing diseases related to oxidative stress.

## Author Contributions

**Meryem Tourabi, Youness El Abdali, Khaoula Faiz, Ibrahim Mssillou, Abdelkrim Agour:** conceptualization, original draft writing, reviewing, and editing. **Bouchra Louasté, Mohammed Merzouki, Ahmad Mohammad Salamatullah, Mohammed Bourhia, Youssouf Ali Younous:** formal analysis, investigations, funding acquisition, reviewing, and editing. **Amira Metouekel, Badiaa Lyoussi, Elhoussine Derwich:** resources, data validation, data curation, and supervision.

## Ethics Statement

The authors have nothing to report.

## Consent

The authors have nothing to report.

## Conflicts of Interest

The authors declare no conflicts of interest.

## Data Availability

All data generated or analyzed during this study are included in this published article.
